# Special Issue “Transcriptomics in the Study of Insect Biology”

**DOI:** 10.3390/ijms252312582

**Published:** 2024-11-22

**Authors:** Yakov E. Dunaevsky, Elena N. Elpidina

**Affiliations:** A.N. Belozersky Institute of Physico-Chemical Biology, Lomonosov Moscow State University, Moscow 119991, Russia; dun@belozersky.msu.ru

Transcriptomics is at the intersection of molecular biology and genetics, and studies the complete set of transcripts that are synthesized in a cell or organism under certain conditions. Transcriptomics is currently one of the fastest growing areas of systems biology, bioinformatics, and medicine, providing information on the functional activity of the genome. Transcriptomic analysis, which aims to identify and quantify the expression levels of various genes, allows us to identify which genes are expressed in a particular tissue of a particular organism at a particular time under certain conditions. Transcriptome data can be used to identify the differences in gene expression between different states of a cell or tissue and, importantly, to study normal and pathological processes [1]. Transcriptomics has already made significant contributions to medical science, allowing us to better understand the molecular basis of various diseases and develop more effective diagnostic and therapeutic methods. For example, it has helped identify molecular markers for breast cancer and has improved our understanding of infertility mechanisms [2,3]. In endocrinology, transcriptomics has helped identify key gene signaling pathways that are involved in the development of diabetes, obesity, and thyroid disease [4]. It has also become an important tool for personalized medicine, allowing therapy to be tailored based on a patient’s specific gene expression profile [5].

Bridging the gap between the genome and phenotype, the transcriptome represents a snapshot of life processes at the molecular level. Transcriptome sequencing is an accurate and efficient deep sequencing technology that serves as a powerful analytical tool, especially for species lacking reference genomic information [6], and has already provided important insights into biological processes in insects. For example, transcriptomics helped in the identification of differentially expressed genes (DEGs) associated with immune defense in the pest of many crops—*Holotrichia parallela*—in response to nematode infestation [7]. Transcriptomic analysis of *H. parallela* larvae in response to EPN-Bt infestation identified important genes encoding antioxidants and detoxifying enzymes [8]. Transcriptomic analysis has been used to uncover functional brain remodeling in insect evolution [9]. Transcriptomic studies provide detailed inventories of the genes involved in generating distinct phenotypes in social species, enabling the identification of key genes and networks involved in creating distinct castes of social insects [10].

The Special Issue “Transcriptomics in the Study of Insect Biology” focuses on the diverse applications and achievements of transcriptomics in basic and applied entomological research in various fields of entomology. It provides a very-high-quality collection of recent research articles covering a wide range of topics, from transcriptome characterization of insect pests, invasive species, and biological control targets to the analysis of insect–microbe and insect–host plant interactions.

The first experimental article in this Special Issue [11] is devoted to the study of the mechanisms underlying the metabolic adaptation of silkworms (*Bombyx mori*) to artificial diets. The natural habit of feeding exclusively on mulberry leaves, developed during evolution, has led to the seasonal nature of silkworm rearing. Inappropriate dietary changes cause maladaptation in silkworms, manifested in growth retardation, reduced immune resistance and a deterioration in silk quality, making the problem of developing an appropriate artificial diet relevant [12]. The studied changes in the gene expression and metabolic composition of Malpighian tubules as an important organ of metabolic excretion and detoxification in silkworms fed with mulberry leaves and artificial diets allowed for the identification of 2436 DEGs and 245 differential metabolites between the groups, with most DEGs being associated with metabolic detoxification, transmembrane transport, and mitochondrial function. RNA sequencing using the Illumina NovaSeq 6000 platform (Illumina, San Diego, CA, USA) allowed for the determination of several detoxification genes that are associated with all three phases of metabolic detoxification; these were significantly upregulated in the Malpighian tubules of silkworms following an artificial diet. It has been suggested that these genes may be used to metabolize the different types of harmful or useless substances that are present in an artificial diet. The increased expression of several transporters during artificial feeding may indicate that the metabolism of substances in the Malpighian tubules of silkworms is severely altered by artificial feeding and, therefore, should be restored. A higher cellular energy requirement was found after artificial feeding. This can be explained by the greater amount of energy required for the transport of various substrates across the biological lipid bilayer, including the removal of toxic substances during metabolic detoxification by ABC transporters or for the transmembrane transport of large amounts of organic solutes and xenobiotics by SLC family transport proteins [13]; this is a process which requires a more active mitochondrial function in the Malpighian tubules to process the components of the artificial diet. Metabolome analysis showed elevated contents of secondary metabolites, terpenoids, flavonoids, alkaloids, organic acids, lipids, and food additives in the artificial diet-fed group. Thus, based on the analysis of the transcriptome and metabolome of silkworm Malpighian tubules, it can be concluded that silkworms adapt to dietary changes by regulating detoxification, transmembrane transport, and mitochondrial functions. Artificial diet affects the growth and development of *Bombyx mori* silkworms, especially at the fifth instar, which may be due to the longest duration and the highest amount of food consumed at this developmental stage; the effects of artificial diet on growth and development may have a cumulative effect. The identification of key DEGs and metabolic components under artificial diet feeding provides new insights into the molecular mechanisms of insect adaptation to different foods and may provide guidance for the optimization of an artificial diet formula for silkworms.

In [14], the molecular mechanisms underlying oviposition in the dark black chafer *Holotrichia parallela*—a pest of agricultural and horticultural crops in China that feeds on a variety of host plants—were investigated. The authors aimed to identify the molecular targets involved in *H. parallela* oviposition in order to develop effective and environmentally friendly pest control methods. RNA-Seq of gonads was used to identify the transcriptomic changes associated with oviposition. Since differences in nutritional value among host plants can affect insect ovaries [15,16] and influence development and reproduction, the effects of feeding adult *H. parallela* on three different plant species were determined. Using a comparative transcriptomic analysis, the authors identified multiple DEGs in *H. parallela* feeding on different host plants; the major signal transduction pathways and genes involved in oviposition were identified. The involvement of the follicle cell protein 3C gene (a critical gene in *H. parallela*) in the regulation of oviposition was confirmed using RNA interference. Thus, by assessing the effects of three host plants on *H. parallela* oviposition, DEGs that may be associated with oviposition and that may potentially be used as targets for pest management were examined. The results expand our understanding of the molecular mechanisms associated with oviposition and provide a method for identifying genes that may be useful in developing pest control strategies.

In [17], transcriptome sequencing and RNA interference were used to elucidate the mechanism of toxicity of natural coumarin on the growth and development of *Spodoptera litura* larvae, which is a pest that damages multiple host plants and causes enormous economic losses. A toxicological assay showed that natural coumarin significantly inhibits the growth and development of *S. litura* larvae. An increase in coumarin concentration resulted in a significant decrease in the weight of cotton bollworm larvae. Using transcriptome sequencing, it was found that 80 and 45 DEGs associated with detoxification were identified in *S. litura* after coumarin treatment from 0 to 24 h and from 24 to 48 h, respectively. Transcriptome analysis showed that the identified differential metabolites within 24–48 h after coumarin treatment were mainly associated with ABC transporters, which play a crucial role in xenobiotic transport [18]. Most of the carboxylesterase-related genes were downregulated from 0 to 24 h after coumarin treatment, while their expression was upregulated from 24 to 48 h. It should be noted that in insects, carboxylesterases are often involved in resistance to organophosphorus compounds, carbamates, and pyrethroids [19]. However, the activity of cytochrome P450 monooxygenase (CYP450) and acetylcholinesterase (AChE) decreased significantly at 48 h after the coumarin treatment of *S. litura*, while glutathione *S*-transferases (GST) activity increased at 24 h. The silencing of the *SlCYP324A16* gene via RNA interference significantly increased *S. litura* larval mortality and decreased individual weight after treatment with coumarin. In addition, the expression of DEGs involved in glycolysis and the tricarboxylic acid (TCA) cycle was inhibited 24 h after coumarin treatment, resulting in a decrease in energy production, while their expression levels increased after 48 h. This suggests that coumarin has a significant effect on physiological metabolism at the early stage (0–24 h) of entry into the midgut, while its effect is attenuated at a late stage (24–48 h).

Based on the obtained results, the authors propose a hypothetical scheme (Figure 1) illustrating the processes occurring after coumarin enters the midgut of *S. litura*. The decrease in AChE and CYP450 activities after coumarin treatment leads to the disruption of coumarin detoxification. However, at the late stage of coumarin action, ABC transporters may be involved in the transport of coumarin to the extracellular space, thereby reducing the toxicity of coumarin against *S. litura*. Thus, the obtained results provide insight into the possible mechanism of coumarin toxicity, laying a foundation for the control of *S. litura*.

In an experimental article [20], transcriptomic profiling was used to study the mechanisms of interaction between two planthoppers that reduce rice yield and are vectors of plant viruses (brown *Nilaparvata lugens* (BPH) and white backed *Sogatella furcifera* (WBPH)), as well as their hosts—two recombinant inbred rice lines with different resistance to these hoppers. The plant–insect interaction is a complex evolutionary system in which an arms race between the two organisms constantly forces them to co-evolve multiple defense responses and develop strategies for their respective survival. The molecular approach to studying the details of plant–insect interactions involved a comprehensive characterization of the transcriptomes of interacting partners during the interaction. Therefore, the results of this work provide a complete picture of the interactions between the planthoppers and rice considered in the context of a previous study [21] on the expression profiles of protective genes in recombinant inbred lines (RILs), obtained by crossing rice varieties RP2068-18-3-5 (resistant to BPH and WBPH) and TN1 (susceptible to BPH and WBPH), after insect infestation. A transcriptome analysis of infected rice genotypes revealed that plants can sense piercing/sucking signals by triggering resistance gene responses and activating downstream mechanisms to enhance resistance to insect attacks; the analysis revealed some differences in plant response to the two planthoppers. In the study published in this Special Issue [20], the authors sought to find out what defense mechanisms BPH and WBPH use in turn to overcome resistance in rice cultivars. Analysis of WBPH RNA-Seq data identified DEGs that may contribute to insect defense when feeding on rice cultivars with different resistance and/or susceptibility to each insect. The differential expression of genes involved in detoxification, digestion, transport, cuticle formation, splicing, and RNA processing was detected. These genes protect insects from host plant secondary metabolites or allelochemicals and promote their survival when feeding on hosts with resistance genes. Moreover, insects feeding on resistant hosts showed a significant enrichment of the transcripts associated with the biological process of apoptosis. A higher expression of sugar transporters, which act as osmoregulators in insects, was observed in both planthoppers feeding on rice cultivars with resistance to either host. Carboxylesterase is an important enzyme in the insect body that detoxifies alkaloids and ferulic acid-based compounds ingested with rice [22]. The study showed a high induction of this gene, which is observed during the initial stages of feeding on BPH-resistant hosts. However, in contrast, only basal expression was observed in BPH feeding on susceptible hosts, suggesting a potential role for this gene in the insect defense mechanism. Another important element in the detoxification process is ABC transporters, which are responsible for the clearance of ingested xenobiotics in insects. In the case of WBPH, a marked upregulation of this gene expression was observed in insects feeding on resistant genotypes, and an almost negligible expression was observed when feeding on susceptible hosts. Protease inhibitors present in host plants inhibit proteases of invading insect pests, resulting in the starvation of insects due to their inability to digest plant proteins, ultimately causing the developmental delay, decreased fertility, and increased mortality of insects [23]. As part of their counter-defense, insects tend to overproduce these enzymes to maintain normal levels of proteolysis in the gut, thereby facilitating the digestion of host proteins and subsequent nutrient absorption. In the current study, it was observed that serine protease gene expression levels in BPH were higher when feeding on resistant hosts. It is hypothesized that a detailed understanding of the molecular strategies adopted by insects to overcome host plant resistance will help develop more effective and robust insect pest control strategies.

The aim of the work [24] was to study several aspects of the interaction between the insect host and the symbiotic bacteria, such as the effect of the presence of cytoplasmically transmitted strains of the bacterium *Wolbachia* on the patterns of gene expression of the insect host *Drosophila melanogaster*, and to examine the possible correlation of these effects with the changes in host fitness and metabolism caused by *Wolbachi*a, as has been described previously using other methods (Figure 2).

A transcriptome approach was used to investigate how two different *Wolbachia* strains affect gene expression in *D. melanogaster* females. Significant changes were detected in the transcriptome of infected flies compared to uninfected flies, as well as in the transcriptome of flies infected with the *Wolbachia* strain wMelCS ^112^ compared to flies infected with the wMelPlus strain, which contained a large chromosomal inversion in the genome and showed increased host tolerance to heat stress [28]. It should be noted that metabolic changes can be considered general for flies infected with *Wolbachia*. This is particularly true for carbohydrate transport and metabolism, for which the changes correspond to increased glucose levels in flies infected with different *Wolbachia* genotypes [27]. Changes in proteolysis can also be considered common to flies infected with different *Wolbachia* strains, which corresponds to changes in food intake in *D. melanogaster* lines infected with two *Wolbachia* genotypes and to the increased resistance to starvation demonstrated for the wMelPlus strain-infected line. Nevertheless, different *Wolbachia* strains altered gene expression differently; the wMelCS ^112^ strain mainly caused a decrease in the expression level of a number of genes (256 genes were downregulated and 94 were upregulated), whereas wMelPlus mainly caused an increase in their expression (122 genes were downregulated and 218 were upregulated). Since the most notable genetic difference between the two strains was a large chromosomal inversion, it is hypothesized that such an inversion could significantly modify the established genetic communications between the host and symbiont. Remarkably, the only stress-related gene upregulated in stress-tolerant flies (infected with wMelPlus) compared to control flies (uninfected and infected with the wMelSC^112^ strain) was the neuropeptide receptor gene corazonin—CrzR—which mediates metabolic, osmotic, and oxidative stress in *Drosophila* [29], and whose role in heat stress tolerance in *Wolbachia*-infected flies remains to be elucidated.

Interspecific hybrid sterility arises as a result of the accumulation of genetic differences between isolated populations of an ancient ancestor [30]. The study of interspecific hybrid incompatibility allows for the reconstruction of speciation mechanisms and the identification of factors that support postzygotic reproductive isolation between closely related species. In [31], the nature of female hybrid sterility in crosses between female *Drosophila melanogaster* and male *D. simulans* was investigated. Using transcriptomic data and molecular, cellular, and genetic approaches, the authors analyzed differential gene expression, transposable element (TE) activity, piwiRNA biogenesis, and the functional defects of oogenesis in hybrids. The premature loss of germ line stem cells was the most prominent oogenesis defect in hybrid ovaries. Despite the identification of a cohort of overexpressed TEs in hybrid ovaries, no evidence was found that their activity could be considered as the main cause of hybrid sterility. The present work revealed a complicated pattern of RNA helicase Vasa expression in the hybrid germline, including the partial targeting of AT-chX piRNA to the *vasa^sim^* allele and a significant zygotic delay in *vasa^mel^* expression. It should be noted that the Vasa protein is a conserved germline marker that is required for piRNA biogenesis and transposon silencing in *Drosophila* [32]. The study described above allows us to conclude that the hybrid sterility phenotype is based on a complex multi-locus genetic and epigenetic differences between the *Drosophila* species used.

Serine endopeptidases (SPs) of the chymotrypsin S1A subfamily are the largest group of peptidases, which play an important role in insect physiological processes such as the digestion, development, and regulation of innate immunity [33]. In [34], SPs were analyzed in the transcriptomes and genomes of the yellow mealworm *Tenebrio molitor,* and their expression profiles were compared at different stages of ontogeny. SP-related proteins of the S1A subfamily found in the transcriptome of *T. molitor* included 269 sequences, of which 137 were identified as active SPs with classical catalytic residues and 125 were annotated as putative inactive SP homologs (SPHs) with one or more substitutions in the catalytic triad. Several groups of active SPs were identified on the basis of their predicted specificity, including trypsins, as well as chymotrypsin- and elastase-like peptidases. Further analysis included the annotation of potential regulatory domains in the propeptide region, phylogenetic analysis to identify evolutionary relationships between SPs/SPHs, and expression profiling of SP and SPH genes in different life stages of *T. molitor*. The results obtained allowed for the identification of SP-associated genes involved in digestion, embryonic development, metamorphosis, and innate immunity, providing valuable information for further physiological, biochemical, and phylogenetic studies of Tenebrionidae beetles. One of the most interesting groups of SPs in *T. molitor* were seven polypeptidases, which showed a complex organization with two or three peptidase units (domains) predicted both as active or inactive, and were expressed mainly in the pupal and adult stages. However, further research is needed to determine the exact structure and functions of such proteins. The data obtained expand our knowledge on SPs/SPHs and provide the basis for further studies investigating the functions of proteins from the S1A subfamily in *T. molitor*.

The aim of the work by the authors of [35] was to obtain the expression patterns of genes specific to individual developmental stages and to annotate key genes that may be essential for the metamorphosis of the potato ladybird *Henosepilachna vigintioctomaculata*, which causes significant economic losses to Solanaceae crops. In this study, high-throughput sequencing was used to analyze the transcriptomes at different developmental stages (egg, larva, pupa, and adult) of *H. vigintioctomaculata*. Several critical pathways associated with specific stages of *H. vigintioctomaculata* development were identified, as well as potential genes for the development of age-specific control strategies. These include the juvenile hormone gene, which regulates developmental stages; the chitinase and chitin synthase genes, indicating the critical role of chitin metabolism at different stages of *H. vigintioctomaculata*; energy production genes at embryonic and pupal stages; and genes related to food digestion and nutrient absorption that are actively expressed in larvae and adults. The results obtained may facilitate the use of targeted genes for pest control. In addition, these data significantly increased the understanding of genomic resources and can serve as a basis for studying developmental and reproductive variation in *H. vigintioctomaculata*.

In summary, the transcriptome analyses presented in this Special Issue helped to answer a number of questions and highlight major areas in entomological science for future research. Most of the articles in this Special Issue are focused on the transcriptomes of insect pests and the identification of genes that may be useful for controlling these pests. Closely related to this topic is the work on the mechanism underlying pesticide resistance. Transcriptomic methods allowed the authors to analyze the interactions of insects and bacteria or insects and host plants in order to identify the most important DEGs of insects as a result of such interactions. Of great interest was the work that, through the identification of key DEGs, provided insight into the molecular mechanisms of insect adaptation to different foods, which provides a theoretical basis for the further optimization of artificial diets, as well as the work aimed at clarifying the molecular basis for the origin of interspecific hybrid sterility. Changes in gene activity during the life cycle of insects were also studied. Overall, the articles in this Special Issue should serve as an impetus for stimulating new research in the field of entomology, and a detailed understanding of the molecular strategies adopted by insects to overcome host plant resistance will help develop more effective and reliable methods of controlling these pests.

## Figures and Tables

**Figure 1:**
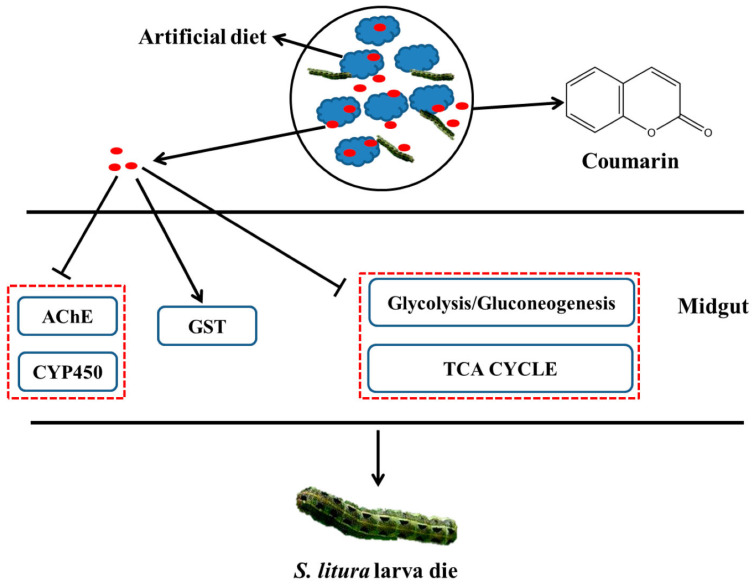
A hypothesized schematic diagram of coumarin effect on enzymes and glycometabolism in *S. litura*.

**Figure 2:**
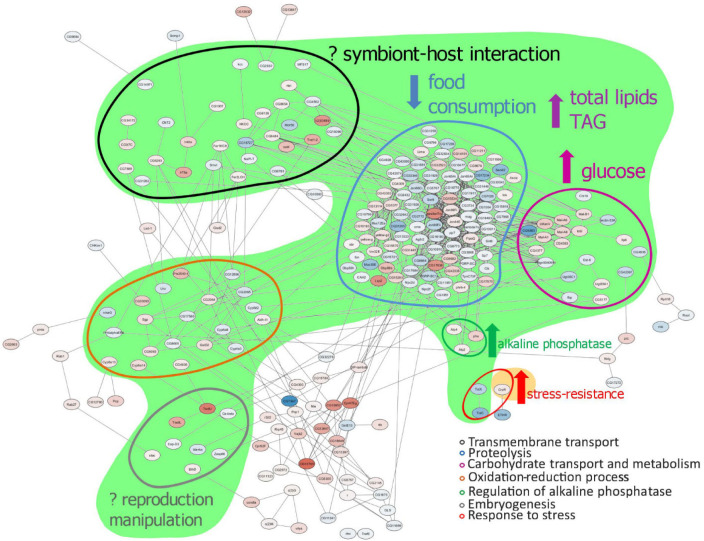
Scheme demonstrating the correlation between the changes in differential expression and the previously shown metabolic and fitness changes caused by *Wolbachia* [25,26,27]. Green region—changes caused by any *Wolbachia* strain; yellow region—changes caused by wMelPlus only. TAG—triglyceride. Upward pointing arrows indicate the increase in a parameter shown in *Wolbachia*-infected flies compared to uninfected ones; the downward pointing arrow indicates the decrease in a parameter shown in *Wolbachia*-infected flies compared to uninfected ones. The question marks indicate possible connection of changes in DEGs and the effect of *Wolbachia* on the host.
